# 1776. Overcoming barriers and expanding an existing Antimicrobial Stewardship Program: Allergy Collaboration to de-label Penicillin allergy

**DOI:** 10.1093/ofid/ofac492.1406

**Published:** 2022-12-15

**Authors:** Ayne Adenew, Angelike P Liappis, Amanda Gillion, Michelle Barcelo, Janine Vanlancker, Dipa Sheth

**Affiliations:** DCVA Medical Center, washington, District of Columbia; DCVA Medical center/The George Washington University, Washington, District of Columbia; Memphis VAMC, Memphis, Tennessee; DCVA medical center, Washington, District of Columbia; DCVA Medical center/The George Washington University, Washington, District of Columbia; DCVA Medical Center/The George Washington University, Washington, District of Columbia

## Abstract

**Background:**

The pandemic placed barriers to face-face visits and repurposed Antimicrobial Stewards. During the pandemic, our existing Antimicrobial Stewardship Program: Allergy PCN or beta-lactam de-labeling program (PDLP) collaborated with a medical center within our network to adopt an existing EMR-based screening tool and CDSS tracking system to overcome barriers.

**Methods:**

Between April 2021-March 2022 we expanded our PDLP by off-loading the workload through EMR beta-lactam assessment (BLA) notes targeting inpatient PharmDs. The ability to de-label was expanded by ‘chart’ delabeling using a BLA note template adopted from the Memphis VAMC. The BLA allowed historical allergy (duration, tolerability of other beta-lactams) or drug-related side effect to be challenged. Referrals to allergy were identified using the BLA; ASP Pharmacists conducted PharmD training and fielded referrals to either de-label with BLA vs PCN testing. BLA notes were tracked over time in CDSS (TheraDoc, DSS Inc) and after evaluation, the EMR was updated to reflect PCN allergy status post-assessment.

**Results:**

Over one year the new BLA note was used in 113 patients; assessments were completed by the targeted audience (Inpatient Medicine PharmD) by the 3^rd^ month of the adoption period; subsequently BLA use increased 180% compared to the 3 month lead-in period. BLAs gave inpatient providers guidance on drug selection, referral for formal PCN-testing or Pharmacist/Allergy review, a “chart only” de-label . With pandemic constraints few patients (17/113, 15%) were referred for skin testing with 65% of those successfully de-labeled. Among those not requiring PCN testing, when EMR records demonstrating tolerability to other beta-lactam agents, non-allergic reactions were clarified and/or patients were well enough during the hospital stay to provide accurate historical information nearly a third of those patients (19/66, 29%) were successfully de-labeled.

**Conclusion:**

The pandemic provided clinical barriers, however we successfully adopted an EMR-based BLA tool. Engagement and buy-in from providers resulted in chart documentation and allergy de-labeling. Allergy working with ASPs can leverage both EMR and CDSS tools to de-label allergies to beta-lactams.

**Disclosures:**

**All Authors**: No reported disclosures.

